# Development and Optimization of Hyaluronic Acid-Poloxamer In-Situ Gel Loaded with Voriconazole Cubosomes for Enhancement of Activity against Ocular Fungal Infection

**DOI:** 10.3390/gels8040241

**Published:** 2022-04-14

**Authors:** Nabil A. Alhakamy, Khaled M. Hosny, Waleed Y. Rizg, Bayan A. Eshmawi, Moutaz Y. Badr, Awaji Y. Safhi, Samar S. A. Murshid

**Affiliations:** 1 Department of Pharmaceutics, Faculty of Pharmacy, King Abdulaziz University, Jeddah 21589, Saudi Arabia; nalhakamy@kau.edu.sa (N.A.A.); wrizq@kau.edu.sa (W.Y.R.); beshmawi@kau.edu.sa (B.A.E.); 2 Center of Excellence for Drug Research and Pharmaceutical Industries, King Abdulaziz University, Jeddah 21589, Saudi Arabia; 3 Department of Pharmaceutics and Industrial Pharmacy, Faculty of Pharmacy, Beni-Suef University, Beni-Suef 62511, Egypt; 4 Department of Pharmaceutics, Collage of Pharmacy, Umm Al-Qura University, Makkah 24381, Saudi Arabia; mybadr@uqu.edu.sa; 5 Department of Pharmaceutics, Faculty of Pharmacy, Jazan University, Jazan 82817, Saudi Arabia; asafhi@jazanu.edu.sa; 6 Department of Natural Products and Alternative Medicine, Faculty of Pharmacy, King Abdulaziz University, P.O. Box 80260, Jeddah 21589, Saudi Arabia; samurshid@kau.edu.sa

**Keywords:** antifungal, hyaluronic acid, Box-Behnken design, voriconazole, nanocubosomes

## Abstract

Fungal eye infections are largely disseminated, especially in developing countries where they may leave over half a million people blind per year. The current study aims to boost the voriconazole antifungal efficiency via loading it as cubosomes (VZ-Cub) into hyaluronic acid and poloxamer-based ocular in situ gel. VZ-Cub were fabricated applying Box-Behnken design and employing phytantriol, poloxamer F127, and VZ amounts as independent variables. The produced nano vesicles were evaluated for the dependent variables of particle size (PS), entrapment efficiency (EE%), and transcorneal steady-state flux (Jss) of the VZ, and, the obtained optimal VZ-Cub was loaded into an in situ gel base to enhance its ocular residence time. The in situ gel formulation was tested for its gelation temperature, drug release behavior, transcorneal permeation effects, and antifungal activity. The optimized VZ-Cub consisted of 100 mg of phytantriol, 60 mg of poloxamer F127, and 21 mg of VZ. This formulation led to a minimum PS of 71 nm, an EE% of 66%, Jss value of 6.5 µg/(cm^2^·min), and stability index of 94 ± 2%. The optimized VZ-Cub-loaded in situ gel released 84% VZ after 12 h and yielded a 4.5-fold increase in drug permeation compared with the VZ aqueous dispersion. The antifungal activity, which was obtained by measuring the fungal growth inhibition zones, revealed that the VZ-Cub-loaded in situ gel formulation had a 3.89-fold increase in antifungal activity compared with the VZ dispersion. In summary, an ocular in situ gel loaded with VZ-Cub could be an effective novel nano-paradigm with enhanced transcorneal permeation and antifungal properties.

## 1. Introduction

Ophthalmic fungal eye infections are highly disseminated in third-world countries causing more than half a million cases of blindness per year [1]. Despite the varying conservative mechanisms protecting the eye against harmful pathogens, several fungal infections that may affect the cornea, conjunctiva, orbit, and other ophthalmic areas are routinely encountered [2]. Dematiaceous fungi, such as *Aspergillus*, *Fusarium*, *Scedosporium*, and *Candida*, are the prevailing species in ocular infections [3]. Sundry of treatment protocols are employed for eye infections based on the diagnosis including surgery, especially for invasive *Fusarium* infections [4]. In addition, several drugs are used in managing eye infections, such as amphotericin B for acute, life-threatening ocular mycoses and natamycin for filamentous fungal keratitis [5,6].

Triazoles that are heterocyclic compounds having 5-membered rings with two carbon and three nitrogen atoms have increasingly grasped attention for treating deep fungal infections avoiding the side effects of the preceding antifungals [7], triazoles, such as fluconazole and itraconazole, have been extensively investigated as therapeutic alternatives for ocular mycoses [8]. 

Voriconazole (VZ) is a robust second-generation triazole that is effective against a wide range of fungi. It is a derivative of the original triazole, fluconazole [9]. VZ disrupts fungal growth via restraining the cytochrome P450 (CYP)-dependent enzyme sterol 14-alpha-demethylase. This deactivates the ergosterol output, which is indispensable for the biosynthesis of fungal cell membranes and can cause fungal cell death [10]. VZ is highly indicated for treating invasive aspergillosis and some other infrequent fungal infections, such as those caused by Scedosporium and Fusarium spp. It has also been recommended for some fungal infections that are resistant to antifungal drugs [11]. However, when VZ is topically introduced as a conventional eye drop, it may have a short residence time and inferior transcorneal permeation owing to ophthalmic defense mechanisms and drug drainage to the nose or gut, so that only minor amounts reach the posterior eye compartment [12,13]. Thus, enhancing its ocular bioavailability depends on improving its residence time within the eye and transcorneal permeation. Many investigators exploring vesicular [14,15,16] and nanoparticulate systems [17,18,19] have confirmed the great ability of these systems to boost the ophthalmic bioavailability of drugs.

Cubosomes are well-advocated novel, lipid-based nanovesicles for delivering drugs because of their minimal toxicity and wide areas of interface between hydrophobic and hydrophilic regions [20]. They are well-stabilized vesicles that are composed mainly of a lipid material within an outer shell containing a polymer [21]. Recently, they have come to the attention of drug formulators because of their ability to boost the solubility and bioavailability of lipophilic drugs [22]. Phytantriol and monoolein are the most prevalent lipid materials employed in cubosomes’ manufacture [23]. Notably, phytantriol displays adequate, biocompatibility, safety, structural stability, and sustained drug release upon using their cubosomes in ophthalmic drug delivery [24]. Using surfactants commonly enhances nanoparticles’ steric and structural stability [25,26]. Non-ionic surfactants, such as pluronics, have been anticipated to reinforce the solubility and stability of inadequately soluble drugs owing to their surface activity and poor toxicity [27]. Poloxamers are well-established steric stabilizers for nanovesicles due to their unique composition; they are non-ionic triblock copolymers constituted from two hydrophilic chains of polyoxyethylene and a hydrophobic chain of polyoxypropylene [28].

In situ gelling systems can be introduced as drops and then switched to a gel at the site of administration and can undergo a sol-to-gel transition [29]. Temperature change, ionic interactions, pH changes, UV inductions, and solvent exchanges are the most common triggers for these systems. As for temperature-triggered gelation, systems are in a liquid state at room temperature and in a gelled state at higher temperatures, and are therefore named thermosensitive gels. Pluronics are the most applied thermosetting polymers and the ones most used for formulating thermosensitive gels [30]. At concentrations of 20% to 23% or higher, poloxamer-F127 is commonly utilized in in situ gels [16]. 

There are some weaknesses of pluronic gels for drug delivery applications, such as weak mechanical properties and minimal residence times due to their rapid dissolution in biological environments [31]. To counteract such limitations, several trials were done in which pluronic gels were chemically changed [32,33,34,35] to obtain in situ gels with adequate mechanical properties. One of these approaches was to mix pluronics with mucoadhesive polymers [36,37,38] that were capable of forming networks via noncovalent bonds with the mucin coating of epithelial cells, hence extending the in vivo residence time of developed formulations. Interestingly, recent scientific investigations paid very special attention to hyaluronic acid (HA), which is a naturally occurring, high-molecular-weight polymer characterized as being biodegradable and strongly biocompatible [39]. HA is a polysaccharide that is a main constituent of the extracellular matrix in connective tissues, and it organizes and has hegemony over several in vivo physiological functions. HA acquires significant viscoelasticity, which can diminish friction on the eye surface when a person blinks to mitigate dry eyes [40] and keratoconjunctivitis [41]. Owing to its viscosity-enhancing, mucoadhesive, and strong mechanical specifications, HA could be a good candidate for incorporation into in situ gelling systems. Further it could be very beneficial to combine the permeation enhancing effect of cubosomes with the outstanding anti-inflammatory, anti-dry eye, and mucoadhesive properties of HA to obtain a novel ocular antifungal formulation of VZ.

Response surface methodology is a well-trusted method for investigating and developing active agents’ delivery systems [42]. It employs various empirical and polynomial designs to harmonize and map the interrelationships of variable tentative scopes. For the current experimental work, the Box-Behnken design (BBD) was used due to its outstanding features, including its rotatable experimental designs for therapeutic associations, the fewer experiments needed for it, and its cost-effectiveness in formulation development [43]. 

To the best of our knowledge, there was no previous investigation exploring the ocular antifungal efficiency of VZ-loaded cubosomes that were further inserted into hyaluronic-acid-based in situ gel. Therefore, the present work was oriented toward developing an in situ thermosensitive gel loaded with VZ cubosomes (VZ-Cub) for enhancing VZ’s transcorneal permeation, ocular residence time, and antifungal activity.

## 2. Results and Discussion

### 2.1. Evaluation of PS and PDI of VZ-Cub

The produced VZ-Cub had a PS of 65 ± 2.5–430 ± 6.0 nm and PDI values ranging from 0.15 to 0.40 (see Table 1), identifying the reasonable homogeneity and uniform size distribution of the VZ-Cub dispersion. 

A linear model of polynomial analysis was used to depict the gathered PS data, as it was found to have the highest mean squared value surpassing the residual error (*p* < 0.0001). The assumed statistical design disclosed the competency of the chosen model to examine the presumed influence of the amounts of phytantriol (A), poloxamer F127 (B), and VZ (C) on the PS. The anticipated R^2^ value (0.8440) of the suggested model was in close agreement with its adjusted R^2^ value (0.8954). An ANOVA analysis of the collected data resulted in Equation (1) below.

(1) PS=+254.12+254.12A −80.98B −13.48C 

**Figure 1:**
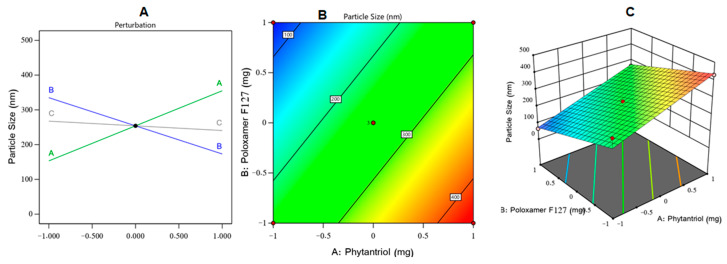
(**A**) Perturbation, (**B**) contour, and (**C**) response surface plots showing the effect of studied independent variables on VZ-Cub particle size.

Figure 1 shows the perturbation diagram, contour, and three-dimensional (3D) surface plot of the PS response. As could be noticed from that figure and the equation above, factor (A) had a significantly positive effect on the PS (*p* < 0.0001), while factor (B) had a significantly negative effect on the same response (*p* < 0.0001) and factor (C) had an insignificant influence on the same dependent variable (*p* = 0.2). The higher PS values of the VZ-Cub were seen following an increase in the amount of phytantriol; this could be due to the small shearing influence and larger potential of cubosome aggregation at elevated phytantriol levels. Comparable outcomes were found in the literature [44].

The decrease in PS values of the VZ-Cub found upon increasing the poloxamer F127 amounts could be explained based on the nature of poloxamer F127. As a non-ionic surfactant, poloxamer F127 can contribute to reducing the interfacial tension between a lipid matrix and the surrounding aqueous phase, resulting in smaller vesicles [45]. Further, poloxamer F127 as a triblock-copolymer could promote steric stabilization via inducing repulsion between individual cubosomes, preventing their aggregation [46].

### 2.2. EE% Assessment

Results listed in Table 1 show that the developed VZ-Cub had a mean EE% of VZ of between 44 ± 2.0% and 88 ± 5.3%. The utilized statistical program proposed a quadratic model of polynomial analysis for EE% value appraisal. The elected model had a *p*-value of less than 0.0001. Hence, the deduced model was capable of evaluating the effect of the amounts of phytantriol (A), poloxamer F127 (B), and VZ (C) on the EE% of the VZ-Cub. The selected model had comparable adjusted and forecasted R^2^ values of 0.9484 and 0.8404, respectively. An analysis of variance of the EE% data yielded the following Equation (2).

(2) $$\begin{array}{ll} \mathrm{EE}\%= & +72.33+9.96A-3.04B-1.79C-2.74\mathrm{AB}-3.74\mathrm{AC}+\\ & 4.76\mathrm{BC}-0.5377A^{2}+1.96B^{2}-16.04C^{2} \end{array}$$

**Figure 2:**
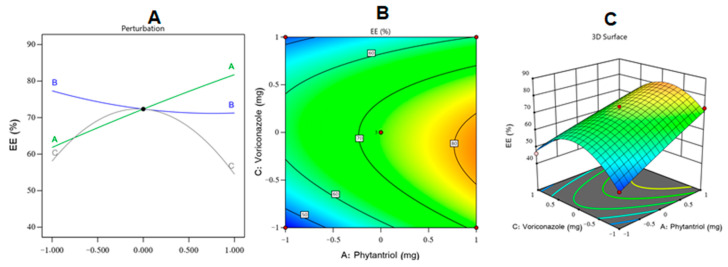
(**A**) Perturbation, (**B**) contour, and (**C**) response surface plots showing the effect of studied independent variables on the EE% of the VZ-Cub.

The obtained findings revealed the predominant influence of phytantriol (*p* < 0.0001) and poloxamer F127 (*p* = 0.0113) on the EE%. Based on the examination of the above equation and Figure 2, which shows the perturbation diagram, contour plot, and 3D surface plot of the EE% response, it was observed that the phytantriol level had the most prominent synergistic action on the EE%. These results could be explained by the ability of phytantriol to form a rigid vesicle bilayer and multilayered cubosomes in addition to the expected high affinity of VZ as a lipophilic drug for phytantriol. Comparable outcomes were detected in ciprofloxacin-loaded phytantriol-based cubosomes and other investigations [44,47,48]. Remarkably, poloxamer F127 exerted a significant antagonistic effect on the EE% of VZ; this might be understood by considering the surface-active property of poloxamer F127, which could reduce the interfacial tension between the cubosomes’ lipid bilayer and the surrounding medium, yielding VZ diffusion into the aqueous medium and lowering the EE% [49,50].

### 2.3. Jss Determination

The Jss of the VZ from the VZ-Cub across the rabbits’ corneas had values of between 4.1 ± 0.22 and 6.9 ± 0.45 µg/(cm^2^·h), as summarized in Table 1.

The best significant average squared value that outweighed the residual error (*p* ˂ 0.0002) was fulfilled by a quadratic model of polynomial analysis, and therefore, it was chosen for analyzing the VZ transcorneal flux data. The endorsed experimental design confirmed the capacity of the selected model to estimate the significant effect of the amounts of phytantriol (A), poloxamer F127 (B), and VZ (C) on the VZ Jss from the VZ-Cub. The allocated model had an adjusted R^2^ value of 0.9517, which was in line with an expected R^2^ value of 0.8632 (Table 2). An ANOVA of the observed data produced Equation (3) that is described below:

(3) $$\begin{array}{ll} \mathrm{Jss}= & +4.42-0.4143A+1.26B+0.1107C+0.0253\mathrm{AB}+0.1753\mathrm{AC}\\ & -0.2247\mathrm{BC}+0.4856A²+0.3356B^{2}+0.1356C^{2} \end{array}$$

**Figure 3:**
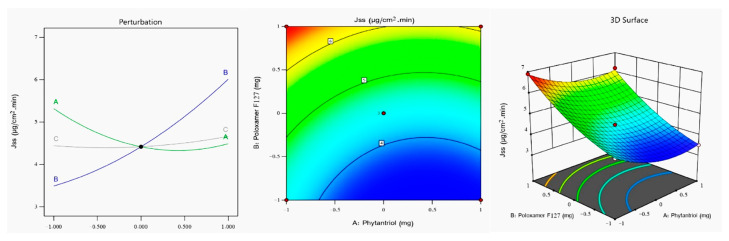
(**A**) Perturbation, (**B**) contour, and (**C**) response surface plots showing the effect of studied independent variables on the Jss of the VZ-Cub.

From the above equation and Figure 3, which illustrates the perturbation plot, contour plot, and 3D surface plot of the VZ Jss, it can be seen that factor (A) had a significant negative effect on the Jss (*p* = 0.001) in contrast to factor (B), which had a significant positive effect on the Jss (*p* < 0.0001) and would largely facilitate VZ permeation across rabbits’ corneas. These outputs could be explained by the PS results; higher levels of phytantriol would result in larger cubosomes offering a smaller surface area for drug release, in addition to increasing the solubility of VZ in the lipid bilayer, and this would decrease the chances of its dispersion into the surrounding medium [51]. On the contrary, poloxamer F127 (B) highly increased the drug’s transcorneal permeation. Such an effect could be due to the permeation enhancement of poloxamer F127, which could be attributed to its ability to change membrane properties by interfering with tear film and mucin, disrupting their protective roles and disorganizing the integrity of the epithelial cell membrane and loosening its tight junctions [52]. Table 2 shows the regression analysis of all measured responses.

### 2.4. Optimization of VZ-Cub

**Figure 4:**
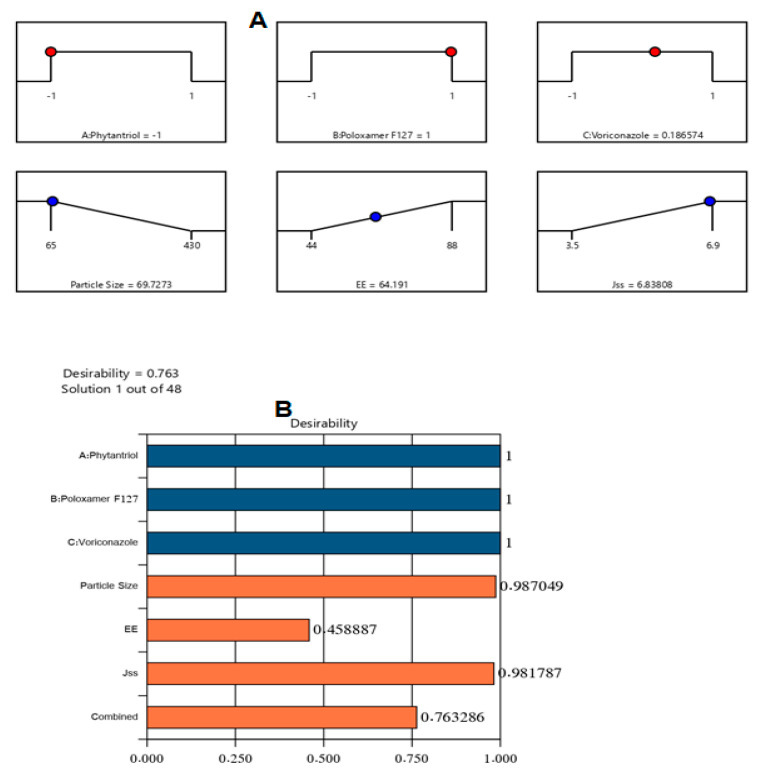
Bar chart and desirability ramp for optimization process. The desirability ramp illustrates the levels of the studied independent factors and the expected values for the measured responses of the optimized VZ-Cub (**A**). The bar chart illustrates the values of desirability for the conjugated responses (**B**).

Upon examining the collected data, an optimal VZ-Cub formulation was made with the most suitable features. Sundry combinations of independent variables were advocated by Design-Expert software. The optimized VZ-Cub formulation had 100 mg of phytantriol, 60 mg of poloxamer F127, and 21 mg of VZ. The fabricated optimized nanocubosome formulation had a PS of 71 nm, EE% of 60%, Jss of 6.5 µg/(cm^2^·h), and ZP of −26.30 ± 0.23 mV with a desirability of 0.763. Figure 4 displays the desirability ramp combining different levels of studied factors and expected values of the measured responses of the optimum formulation. Table 3 shows that the real values of the optimal formulation’s responses were very close to the expected ones with no high variations (*p* > 0.05), proving the model’s adequacy, validity, and precision.

**Figure 5:**
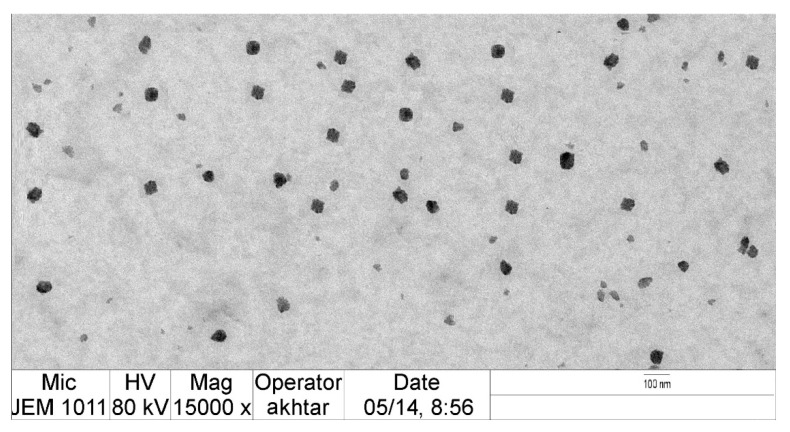
TEM image of optimized VZ-Cub formulation.

The optimized VZ-Cub formulation was distinctly cubical in shape with smooth surfaces, as recorded by TEM (Figure 5).

The tested optimized formulation passed the heat–cool test with no significant signs of instability. The stability index of the VZ-Cub is a parameter of paramount importance in assessing their stability. The VZ-Cub exhibited stability index value 94% ± 2. These values are quite reasonable and make the VZ-Cub quite stable.

### 2.5. Rheological Characterization of Optimal VZ-Cub-Loaded In Situ Gel

**Figure 6:**
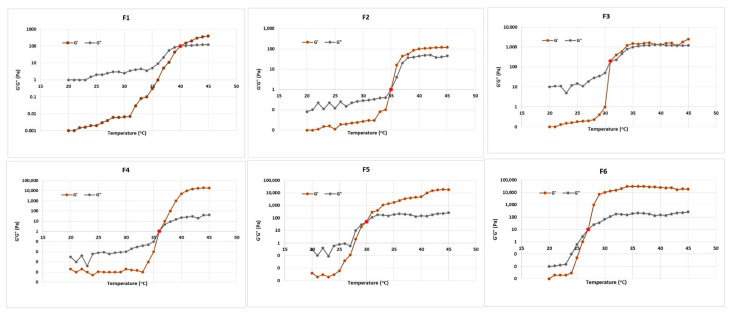
Elastic (G′) and viscous (G″) moduli of poloxamer F127/HA polymers in situ gel as a function of the temperature at a frequency value of 0.01 Hz. Results are the means of three measurements. SD was always lower than 10%. Error bars are omitted for clarity.

Observing the changes in the viscoelastic parameters of used polymers with temperature was the basis for the gelation process evaluation. The viscous and resilient moduli of poloxamer F127 and HA relative to temperature, at a frequency of 0.01 Hz, are illustrated in Figure 6. The temperature of gelation (T_gel_) was recognized as the point at which the G″ and G′ curves met each other. T_gel_ values of varying thermosensitive in situ gel formulations are presented in Table 4. 

The purpose of this study was to develop a semisolid dosage form that was both mucoadhesive and thermosensitive; therefore, poloxamer F127 was combined with HA to attain the required characteristics. The effect of HA on pluronic gelation was determined for formulations containing 0.2% and 0.4% *w*/*w* of HA. The used HA did not impede the gelation process of the poloxamer F127 and only resulted in lowering its gelation temperature. The addition of 0.2% HA to the used concentrations of poloxamer F127 resulted in a decrease in the T_gel_ from 40 (F1) to 31 °C (F3), while F2 had a gelation temperature of 35 °C. Adding 0.4% HA resulted in lowering of the T_gel_ from 36 (F4) to 27 °C (F6). Consequently, formulations F2 and F4 were used for further investigation due to their suitable gelation temperatures, which were close to that of the human body. 

### 2.6. In Vitro Release Studies

**Figure 7:**
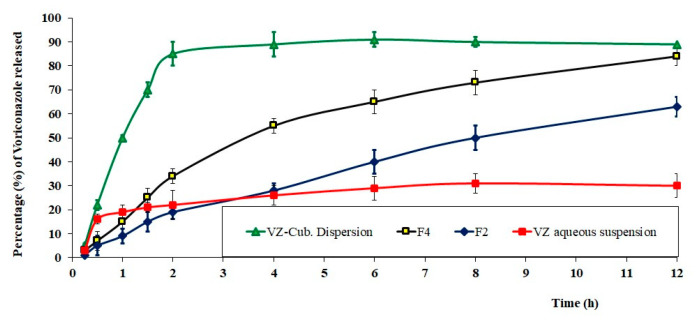
In vitro release profiles of VZ from optimal VZ-Cub dispersion, F4 and F2 in situ gels, and VZ aqueous suspension (mean ± SD, n = 3).

The VZ release percentage from the studied formulations varied as follows: F2 released 63%, F4 released 84%, the VZ aqueous dispersion released 30%, and the VZ-Cub aqueous dispersion released 89% of the VZ after 12 h. Figure 7 shows the in vitro release profiles of the studied formulations. As can be noticed, all formulations that contained the drug in the form of a nanodispersion (either the Cub dispersion, F2, or F4) enhanced the dissolution and release profile of VZ compared with the VZ aqueous dispersion. Notably, the two studied in situ gel formulations (F2 and F4) showed a controlled release for VZ compared with the optimal VZ-Cub dispersion, which released its maximum amount within only 2 h. This means that the optimal VZ-Cub formulation needs to be applied more frequently than the controlled release in situ gel formulations. The viscosity of formulation F2 was higher than that for F4 because F2 contained more poloxamer F127 (15%) than F4 (10%). F4 was selected for subsequent tests due to its high release percentage and controlled release profile compared with all of the tested formulations.

### 2.7. Ex Vivo Transcorneal Permeation Studies

Table 5 shows the ex vivo permeation results of the optimized VZ-Cub nanodispersion, VZ aqueous dispersion, and in situ gel formulation F4 via the corneal membrane. All tested formulations had significant differences in all the measured parameters. The ex vivo permeation findings revealed that the percentage permeated was 76% for F4, 41% for the VZ-Cub nanodispersion, and 16.5% for the VZ aqueous dispersion. These results clarify that formulation F4 enhanced the VZ permeation through the cornea by more than 4.5-fold compared with the aqueous dispersion of the drug.

One major explanation for this permeation enhancement might be either the surface lipid exchange, absorption between the nanocubosomes and ocular epithelial cells, or in combination. The strong adhesion of the minute lipid colloidal nanocarriers with their large surface areas to the lipophilic epithelium cell membrane could boost the VZ permeation in its unionized form via the corneal epithelium and supply more drug to the anterior eye [53]. Moreover, poloxamer F127 is well known for its penetration enhancement effect due to its surface-active property, which could contribute to fluidizing the ocular phospholipid membrane and lead to greater drug penetration [54]. Further, the in situ gel formulation could increase the ocular residence time and offer longer periods for permeation to take place. Analogous results were reported in the literature [16].

### 2.8. Assessment of Antifungal Activity of VZ-Cub-Loaded In Situ Gel

The diameters of the inhibition zones of F4, the VZ-Cub nanodispersion, and the VZ aqueous dispersion were 25.3 ± 2.2, 17.3 ± 1.9, and 6.5 ± 0.8 mm, respectively. This shows that formulating VZ as a nanocubosome enhanced the antifungal activity by approximately 2.66-fold compared with the VZ aqueous dispersion. The incorporation of the optimal VZ-Cub into an HA-poloxamer in situ gel base caused even further enhancement in the antifungal activity (i.e., >3.89-fold) compared with the free drug. Such outcomes also indicate that the presence of HA in F4 promoted the antifungal activity by 1.46 times compared with the VZ-Cub aqueous dispersion that contained no HA. Such a finding could be ascribed to the antifungal activity of HA against several fungal strains, as was reported by other researchers [55]; the mechanism of such an effect remains to be explored.

## 3. Conclusions

In the current treatise, BBD was found to be a beneficial tool for designing and optimizing VZ-Cub. VZ could be efficiently incorporated into nanocubosomes with acceptable specifications. The fabricated optimal VZ-Cub formulation had an average PS value of 71 nm, EE% of 66%, and Jss of 6.5 µg/(cm^2^·h), which might be considered very satisfactory outcomes. The in situ gel formulations F2 and F4 exhibited acceptable gelation temperatures of 35 °C and 36 °C, respectively. Further, the optimized VZ-Cub-loaded in situ gel formulation had enhanced in vitro drug release and ex vivo permeation in contrast with optimal formulation dispersion and drug aqueous suspension. Finally, the optimum formulation loaded into the in situ gel had the largest growth inhibition zones compared with VZ-suspension and optimal VZ-Cub dispersion. This affirmed its excellent antifungal activity, which could be due to the addition of the antifungal action of HA to that of VZ. Thus, this exploration confirms that the VZ-Cub-loaded in situ gel might be an effective combination that offers an outstanding paradigm to boost VZ release and transcorneal permeation along with utilizing the anti-inflammatory, anti-dry eye, and mucoadhesive properties of HA to achieve an optimum platform in managing ocular fungal infections.

## 4. Materials and Methods

### 4.1. Materials

VZ, phosphate buffers, and methanol were purchased from Sigma Chemical Company (St. Louis, MO, USA). HA was supplied by Fidia (Abano Terme, Padua, Italy). Phytantriol (3,7,11,15-tetramethyl-1,2,3-hexadecanetriol) was purchased from Avanti Polar Lipids (Alabaster, AL, USA). Poloxamer F127 (MW 12,600 g/mol) was purchased from BASF (Ludwigshafen, Germany). Acetonitrile for high-performance liquid chromatography (HPLC) gradient grade analysis was purchased from Fisher Scientific (Loughborough, Leicestershire, UK). All other reagents and chemicals used were of analytical grade. 

### 4.2. Methods

#### 4.2.1. Experimental Design

In this investigation, the BBD was followed to match investigated factors and measured responses. A design for 17 formulations was generated using Design-Expert Version 13 software (Stat-Ease, Inc., Minneapolis, MN, USA). The design provides varying combinations of factor levels, as shown in Table 1. The three independent variables examined were the amounts of phytantriol (A) (100, 150, and 200 mg), poloxamer F127 (B) (20, 40, and 60 mg), and VZ (C) (15, 20, and 25 mg). In the same context, the particle size (PS, Y_1_), entrapment efficiency (EE%, Y_2_), and steady-state flux (Jss, Y_3_) were the evaluated dependent variables. Factor levels were selected based on preliminary studies before applying the proposed experimental design to obtain the best combinations that would produce nanocubic vesicles. 

#### 4.2.2. VZ-Cub Preparation 

Based on combinations suggested from the BBD, 17 VZ-Cub formulations were fabricated using the top to bottom method [56]. Phytantriol was used in range of 100–200 mg as (A), poloxamer F127 was added in amounts ranging from 20 to 60 mg as (B), and VZ was added in amounts ranging from 15 to 25 mg as (C). In brief, for the VZ-Cub preparation, varying phytantriol quantities were weighed, transferred into glass vials, and heated to 45 °C until they were freely flowing. Then, known amounts of VZ and poloxamer F127 (Table 2) were mixed with phytantriol in 10 mL of phosphate buffer (PBS, pH 7.4). The obtained mixture was homogenized using ULTRA-TURRAX (T25 digital, IKA-Werke GmbH & Co. KG, Staufen, Germany) at 15,000 rpm for 5 min at 45 °C. This methodology continued until a milky dispersion was attained. 

#### 4.2.3. Determination of Particle Size and Polydispersity Index of VZ-Cub 

When formulations were ready, the particle size (PS) and polydispersity index (PDI) were determined using a Zetatrac particle size analyzer (Microtrac, Inc., Montgomeryville, PA, USA) at the ambient temperature. Briefly, 100 µL of the VZ-Cub dispersions were diluted using 900 µL of PBS (pH 7.4) and mixed for 3 min. Then, 100 μL of the diluted samples was disengaged and examined for PS and zeta potential (ZP). Measurements were performed three times, and results were calculated as the mean of the performed tests (n = 3, means ± SD) [57].

#### 4.2.4. Determination of VZ EE%

A direct method was used to assess the EE% of VZ. In short, certain amounts of each of the 17 cubosomal dispersions were centrifuged at 15,000 rpm for 30 min at 4 °C (Beckman Coulter, Inc., Fullerton, CA, USA). The supernatant was removed, leaving the cubosomal pellets, which were sonicated with methanol for 15 min. The concentration of VZ was attained by a spectrophotometer at 261 nm (UV-1601 PC, Shimadzu Corp., Kyoto, Japan). The EE% was calculated based on the following Equation (4):

EE% = (ME/MT) × 100(4)

where, ME and MT are the quantities of encapsulated and total VZ, respectively [58].

#### 4.2.5. Ex Vivo Permeation Study (Jss Measurement)

The release behavior and permeation parameters of the developed VZ-Cub were evaluated by performing an ex vivo permeation analysis. These parameters included the steady-state flux (Jss), which was an independent variable in the BBD. To obtain corneas for the transcorneal permeation membrane, albino rabbits’ eyes (51 rabbits/3 rabbits for each formulation) were enucleated after the rabbits were humanely killed with intravenous excess sodium phenobarbital. Corneas were then dissected according to a previously described procedure [59]. The method agreed with the ethical principles of the Egyptian Research Institute of Ophthalmology for animal use in experiments (approval No. 00136-1/22). The dissected corneal membranes were inserted in the Franz diffusion cell with 1 cm^2^ surface area, in the donor chamber of a Franz diffusion cell apparatus (Hanson Research, Chatsworth, CA, USA) for 12 h at 37 °C, while the receptor chamber was filled with 7 mL of PBS (pH 7.4).

To assess different permeation parameters of the developed formulations, samples were withdrawn at predetermined time intervals of 0.5, 1, 2, 3, 4, 5, 6, and 12 h and VZ concentrations were determined using a previously described HPLC method. The HPLC method used a column: Phenomenex luna C18 (250 × 4.6 mm, 5 mL); mobile phase: 55/45; organic phase (41/18/10 methanol/acetonitrile/tetrahydrofuran)—buffer (2.5 mmol L^−1^ EDTA-2Na); flow rate: 1.0 mL min^−1^; column temperature: 30 °C; detection wavelengths: 383 and 303 nm; and injection volume: 20 µL. The cumulative amount permeated per unit area (Q) was directly determined from the concentration of drug in the receptor compartment of the Franz diffusion cell apparatus. The Jss was determined from the slope of the steady-state portion of a plot of Q versus time. All experiments were conducted three times, and data were presented as the mean ± SD.

#### 4.2.6. Statistical Analysis of Box-Behnken Design 

Data collected from evaluating the VZ-Cub according to the PS, EE%, and Jss were used to determine the statistical significance of the relationships between variables and responses. Differences among data were considered significant when the *p*-value was less than 0.01. The minimum PS, maximum EE%, and maximum Jss were the bases for selecting the optimal VZ-Cub formulation.

#### 4.2.7. Preparation and Characterization of the Optimized Formulation

The optimal VZ-Cub formulation was fabricated using 100 mg of phytantriol, 60 mg of poloxamer F127, and 21 mg of VZ as suggested by the software. It was then characterized for all parameters, including the PS, EE%, Jss, and ZP. Differences between real and tentative values were also calculated. Transmission electron microscopy (TEM) was used to examine the surface morphology of optimized VZ-Cub formulation. The stability of VZ-Cub formulation evaluated through three sequential freeze–thaw cycles (freezing at −25 °C for approximately 12 h and then, thawing at 25 °C for another 12 h). After these cycles, the final sample was analyzed for droplet size. The sample stability index was determined by comparing the obtained droplet size with that in initial measurements, using Equation (5).

Stability index = ((Initial size − Change in size)Initial size) × 100(5)

#### 4.2.8. In Situ Gel Preparation

Different amounts of poloxamer F127 were dissolved in water by sonication at 4 °C to make a clear polymeric solution containing 10%, 15%, and 20% *w*/*w* poloxamer F127. Then, the HA was added to the pluronic mixtures at room temperature in different amounts to obtain concentrations ranging between 0.2% and 0.4% *w*/*w*. Following that, 5 mL of the optimum VZ-Cub formulation was rigorously mixed with the formed polymer solution until a homogenous mixture was reached. The amounts of poloxamer F127 and HA used were based on preliminary studies and a literature review.

#### 4.2.9. Evaluation of VZ-Cub-Loaded In Situ Gels

##### Rheological Characterization

The rheological behavior of the prepared gels was defined by the small-amplitude oscillatory shear method with a rotational rheometer. The procedures were carried out at different temperatures (i.e., 25 and 45 °C) with an oscillation frequency ranging from 0.1 to 10 Hz and a strain amplitude at which linear viscoelasticity was attained. Thus, the shear storage or elastic modulus (G′) that gave information about the elasticity, or the energy stored in the material during deformation, as well as the shear loss or viscous modulus (G″) that depicted the viscous character, or the energy lavished as heat was measured as a function of frequency was obtained. 

The change in the viscous and elastic moduli with temperature (i.e., from 25 to 45 °C) was detected in order to investigate the gelation temperature of the polymeric systems. This procedure was done at a fixed frequency of 0.01 Hz so as to inhibit any effect on the gelation process and at a strain amplitude where the linear viscoelasticity was valid. The gelation temperature was recognized as the temperature at which the sample switched from a predominantly viscous behavior (G″ > G′) to a prevailing elastic behavior (G′ > G″). Table 4 clarifies the composition of the developed in situ gel formulations.

##### In Vitro Release Studies

The VZ released from different formulations (F2 in situ gel, F4 in situ gel, aqueous dispersion of VZ, optimal VZ-Cub dispersion) was estimated using dialysis bags. They were filled with 5 mL of each tested formulation and kept in a vessel holding 100 mL of PBS (pH 7.4); the dissolution medium was kept at 37 ± 1 °C and stirred at 50 rpm. Samples were withdrawn at certain intervals for a period of 12 h, and the release medium was replenished with an equal volume of fresh medium after every sample withdrawal. Withdrawn samples were analyzed for VZ content at 261 nm using the previously stated HPLC method. Measurements were done three times.

##### Ex Vivo Transcorneal Permeation Studies

A permeation analysis was performed to assess the permeation parameters of the VZ in different formulations, that is, the VZ aqueous dispersion, VZ-Cub aqueous dispersion, and optimized VZ-Cub in situ gel (F4), using the procedure previously mentioned in Section 4.2.5. The determined parameters were the cumulative amount permeated (μg/cm^2^), steady-state flux (Jss) (μg/(cm^2^·min)), diffusion coefficient (D) (cm^2^/min), enhancement ratio (ER), and permeability coefficient (cm/min).

##### Antifungal Activity Assessment

A modulated previously reported disk-diffusion susceptibility procedure [60] was adopted to test the antifungal activity of the optimized VZ-Cub in situ gel formulations against *Aspergillus flavus*. According to the guidelines of the Clinical and Laboratory Standards Institute, an *A. flavus* suspension was made using the 0.5 McFarland turbidity standard (10^6^ CFU/mL) using a hemocytometer. Sterile loops were used to apply an inoculum of 1 mL from a suspension of 10^4^ CFU/mL on Mueller–Hinton agar plate surfaces. Disks of 10 mm diameter that were immersed in different formulations, that is, the optimized VZ-Cub in situ gel (F4), VZ-Cub nanodispersion, and VZ aqueous dispersion, were subsequently placed over the inoculated plates using sterile forceps. The provided Petri dishes were incubated for 24 h at 37 °C, and finally, the diameters of the fungal growth inhibition zones were measured for each formulation using a caliber.

## Figures and Tables

**Table 1:** The composition of different VZ-Cub formulations as determined by the BBD, indicating values for each independent variable (A, B, and C) and the measured responses (Y_1_, Y_2_, and Y_3_).

	A	B	C	Y_1_	Y_2_	Y_3_	
Run	Phytantriol	Poloxamer F127	Voriconazole	Particle Size	EE	Jss	PDI
	(mg)	(mg)	(mg)	(nm)	(%)	µg/(cm^2^·min)	
1	200	20	20	430 ± 6.0	88 ± 5.3	3.5 ± 0.50	0.17
2	150	20	15	350 ± 6.5	69 ± 3.1	3.5 ± 0.22	0.22
3	100	40	15	140 ± 5.8	44 ± 2.0	5.8 ± 0.61	0.19
4	150	20	25	260 ± 8.0	55 ± 1.9	4.1 ± 0.44	0.15
5	100	20	20	290 ± 6.6	67 ± 3.8	4.4 ± 0.31	0.34
6	150	60	25	180 ± 2.9	57 ± 4.1	5.9 ± 0.24	0.40
7	100	40	25	150 ± 5.2	46 ± 2.2	5.4 ± 9.62	0.39
8	200	40	25	350 ± 9.9	60 ± 5.1	4.7 ± 0.41	0.38
9	100	60	20	65 ± 2.5	65 ± 5.4	6.9 ± 0.45	0.33
10	150	60	15	165 ± 3.5	52 ±2.7	6.1± 0.33	0.35
11	200	40	15	400 ± 10	73 ± 6.6	4.3 ± 0.18	0.40
12	150	40	20	240 ± 7.2	72 ± 5.9	4.3 ± 0.27	0.29
13	150	40	20	255 ± 6.5	71 ± 7.0	4.5 ± 0.41	0.19
14	150	40	20	220 ± 4.5	74 ± 6.9	4.4 ± 0.42	0.26
15	200	60	20	265 ± 8.1	75 ± 5.7	6.2 ± 0.39	0.38
16	200	60	25	290 ± 5.9	64 ± 3.9	6.5 ± 0.51	0.37
17	100	20	15	270 ± 7.1	48 ± 3.3	4.1 ± 0.22	0.25

**Table 2:** Regression analysis results for Y_1_, Y_2_, and Y_3_ responses.

	R^2^	Adjusted R^2^	Predicted R^2^	SD	CV%	Adeq. Precision
Response Y_1_	0.915	0.8954	0.844	31.1	12.24	24.1108
Response Y_2_	0.9774	0.9484	0.8404	2.72	4.28	21.9001
Response Y_3_	0.9789	0.9517	0.8851	0.2349	4.72	19.9592

**Table 3:** Actual and experimental values of the optimized VZ-Cub formulation.

	Phytantriol (mg)	Poloxamer F127 (mg)	VZ (mg)	Particle Size (nm)	EE (%)	Jss (µg/cm^2^·h)	Desirability
Predicated value	100	60	21	69.7	64.19	6.83	0.763
Experimental value	100	60	21	71	60	6.5	0.763

**Table 4:** Gelation temperature of different VZ-Cub-loaded in situ gel formulations (mean ± SD, n = 3).

Formulation	Poloxamer Conc. (% *w*/*w*)	Hyaluronic Acid Conc. (% *w*/*w*)	Tgel
F1	10%	0.2%	40 ± 0.5 °C
F2	15%	0.2%	35 ± 0.2 °C
F3	20%	0.2%	31 ± 0.3 °C
F4	10%	0.4%	36 ± 0.2 °C
F5	15%	0.4%	30 ± 0.1 °C
F6	20%	0.4%	27 ± 0.4 °C

N.B. all formulations contain 0.3% *w*/*v* of VZ.

**Table 5:** Permeation parameters.

Parameters of Permeation	F4	VZ-Cub Aqueous Dispersion	VZ Aqueous Dispersion
Cumulative amount permeated (μg/cm^2^)	1741 ± 201	939 ± 113	379 ± 52
Steady-state flux, Jss, (μg/cm^2^·min)	13.21 ± 1.1	6.5 ± 0.3	1.7 ± 0.2
Permeability coefficient, Pc, (cm/min)	12.3 × 10^−4^	7.6 × 10^−4^	3.2 × 10^−4^
Diffusion coefficient, D, (cm^2^/min)	33.2 × 10^−5^	18.4 × 10^−5^	7.1 × 10^−5^
Enhancement factor (EF)	4.59	2.477	-

## Data Availability

Not applicable.
